# Effects of whey and soy protein supplementation on inflammatory cytokines in older adults: a systematic review and meta-analysis

**DOI:** 10.1017/S0007114522001787

**Published:** 2023-03-14

**Authors:** Konstantinos Prokopidis, Mohsen Mazidi, Rajiv Sankaranarayanan, Behnam Tajik, Anne McArdle, Masoud Isanejad

**Affiliations:** 1 Department of Musculoskeletal Biology, Institute of Life Course and Medical Sciences, University of Liverpool, Liverpool, UK; 2 Nuffield Department of Population Health, Medical Sciences Division, University of Oxford, Oxford, UK; 3 Liverpool Centre for Cardiovascular Science, University of Liverpool, Liverpool Heart & Chest Hospital, Liverpool, UK; 4 Institute of Public Health and Clinical Nutrition, University of Eastern Finland, Kuopio, Finland

**Keywords:** Whey protein, Soy protein, Inflammation, IL-6, TNF-α, Sarcopenia

## Abstract

**Background and aims:**

Low-grade inflammation is a mediator of muscle proteostasis. This study aimed to investigate the effects of isolated whey and soy proteins on inflammatory markers.

**Methods:**

We conducted a systematic literature search of randomised controlled trials (RCT) through MEDLINE, Web of Science, Scopus and Cochrane Library databases from inception until September 2021. To determine the effectiveness of isolated proteins on circulating levels of C-reactive protein (CRP), IL-6 and TNF-α, a meta-analysis using a random-effects model was used to calculate the pooled effects (CRD42021252603).

**Results:**

Thirty-one RCT met the inclusion criteria and were included in the systematic review and meta-analysis. A significant reduction of circulating IL-6 levels following whey protein [Mean Difference (MD): −0·79, 95 % CI: −1·15, −0·42, I^2^ = 96 %] and TNF-α levels following soy protein supplementation (MD: −0·16, 95 % CI: −0·26, −0·05, I^2^ = 68 %) was observed. The addition of soy isoflavones exerted a further decline in circulating TNF-α levels (MD: −0·20, 95 % CI: −0·31, −0·08, I^2^ = 34 %). According to subgroup analysis, whey protein led to a statistically significant decrease in circulating IL-6 levels in individuals with sarcopenia and pre-frailty (MD: −0·98, 95 % CI: −1·56, −0·39, I^2^ = 0 %). These findings may be dependent on participant characteristics and treatment duration.

**Conclusions:**

These data support that whey and soy protein supplementation elicit anti-inflammatory effects by reducing circulating IL-6 and TNF-α levels, respectively. This effect may be enhanced by soy isoflavones and may be more prominent in individuals with sarcopenia.

Ageing is associated with increased levels of circulating pro-inflammatory cytokines such as C-reactive protein (CRP), interleukin-6 (IL-6) and tumour necrosis factor-alpha (TNF-α)^(1)^, which are forerunners of cellular senescence and muscle proteolysis^(2)^.

Accruing adverse changes in muscle physiology across the lifespan may lead to reduced muscle mass and physical capacity, particularly after the fifth decade^(3)^, known as sarcopenia^(4)^. From the beginning of the fourth decade, muscle mass decreases by approximately 0·5 % every year. The multifactorial determinants of this phenomenon include reduced levels of anabolic hormones, chronic inflammation, degradation of the muscle contractile proteins, loss of regenerative capacity, altered neural activation, and mitochondrial dysfunction^(5,6)^. Sarcopenia is associated with an increased circulating pro-inflammatory signalling (i.e., higher levels of TNF-α and IL-6)^(7,8)^. In conjunction with sarcopenia, concomitant accumulation of adiposity has also been observed during ageing, representing sarcopenic obesity, which is also linked with elevated inflammatory markers^(9,10)^. Accelerating age-related muscle wasting is partially explained through systemically and locally elevated oxidative stress and reactive oxygen species (ROS) accumulation^(11–13)^. Excessive ROS levels may result in damaged muscle and DNA proteins, triggering the release of pro-inflammatory cytokines and leading to low-grade inflammation^(14)^. Interestingly, antioxidative properties derived from nutrients may prevent excess ROS inflation that could alter muscle proteostasis^(15)^. Hence, finding nutritional strategies to mitigate low-grade inflammation may be considered as a safe and effective strategy for the prevention and treatment of sarcopenia.

Albeit protein supplementation is associated with reduced circulating levels of pro-inflammatory cytokines^(16,17)^, different protein sources may exert distinct anti-inflammatory effects^(18)^. Specifically, soy food intake has been associated with lower circulating levels of IL-6 and TNF-α^(19)^; however, the functional properties of whole foods may differ compared with nutrients in isolation^(20)^. In this regard, previous systematic reviews have observed a reduction of serum CRP levels following intact whey and soy protein supplementation^(21,22)^, while the addition of soy isoflavones has been linked with a decline in circulating IL-6 levels among postmenopausal women^(23)^. Thus, isolated sources of protein may elicit promising isolated anti-inflammatory responses, although the most effective source of intact protein in alleviating circulating pro-inflammatory cytokine levels remains to be fully elucidated. To date, no previous meta-analysis has investigated the effects of intact whey and soy protein supplementation on multiple inflammatory markers in older adults. The aim of this systematic review and meta-analysis is to investigate the effects of intact whey and soy protein supplementation on serum CRP, IL-6 and TNF-α levels in older adults.

## Methods

This systematic review and meta-analysis was conducted in accordance with the Preferred Reporting Items for Systematic Reviews and Meta-Analyses (PRISMA) guidelines^(24)^. The protocol of this systematic review and meta-analysis was registered in the PROSPERO International prospective register of systematic reviews (CRD42021252603).

### Search strategy

Two independent reviewers (KP and MI) searched the MEDLINE, Web of Science, Scopus and Cochrane Library databases from inception until September 2021, using the following search terms: ‘whey OR soy’ in combination with ‘older adults’ and ‘inflammation OR high sensitivity-C reactive protein OR C reactive protein OR IL-6 OR tumour necrosis factor-a’. The complete search strategy is presented in Supplementary Table 1. No restrictions in terms of geographical region were applied. Articles were written in English and discrepancies in the literature search process were resolved by a third investigator (MM).

### Study selection

Studies in this systematic review and meta-analysis were included based on the following criteria: (1) they were RCT; (2) the intervention group received intact soy or whey protein supplements in oral form; (3) the comparator group received a placebo or a non-identical appropriate treatment; (4) circulating levels of CRP, IL-6 and/or TNF-α were assessed; (5) participants that took part in the intervention had a mean age ≥ 50 years old and (6) full text was written in English. Accordingly, studies were excluded if: (1) they were not randomised trials; (2) participants were institutionalised; (3) studies were missing the baseline and/or post-intervention outcome values; (4) whey and soy protein products were in peptide/whole-food form and (5) whey and soy protein supplements were consumed enterally (Supplementary Table 2). Finally, if studies were comprised of a comparator group of < 50 years of age, they were included in the analysis as long as the participant age was similar to the intervention group.

### Data extraction and quality assessment

Two authors (KP and MI) extracted data independently on name of first author, date of publication, country of origin, study design, participant health status, gender, age, BMI, sample size, intervention type, dose and duration, daily energy and protein intake, serum high-sensitivity CRP (hs-CRP), CRP, IL-6 and TNF-α levels. CRP and hs-CRP units were converted to mg/l, while IL-6 and TNF-α values to pg/ml. Disagreements between authors on data eligibility were resolved by a third reviewer (MM). When studies contained multiple doses of protein supplementation, only the highest dose was considered as the intervention arm.

The quality of included studies was evaluated using the Risk-of-bias 2 tool and the Grades of Recommendation, Assessment, Development and Evaluation (GRADE) system approach. Risk-of-bias 2 is a detailed and comprehensive tool to assess the risk of bias in randomised trials included in Cochrane Reviews, focussing on (1) the evaluation of randomisation process, (2) deviations from intended interventions, (3) missing outcome data, and (4) measurement of the outcome and selection of the reported result^(25)^. According to the Risk-of-bias 2 scoring system, study quality was defined as high, some concerns or low. Additionally, the GRADE approach involves the consideration of (1) within-study risk of bias, (2) directness of evidence, (3) study heterogeneity, and (4) precision of effect estimates and risk of publication bias, using four levels of quality (high, moderate, low and very low)^(26)^.

### Data synthesis and statistical analysis

Our analysis reported on the differences among circulating inflammatory markers (hs-CRP, CRP, IL-6, and TNF-α) following whey and soy protein supplementation, when compared with individuals receiving placebo or a non-identical treatment. Quantitative data were treated as continuous measures and were combined by calculating the mean differences between outcomes from baseline and the follow-up period of each intervention. Statistical significance between the intervention and comparator groups was assessed using the random effects inverse-variance model. Missing standard deviations of outcomes were estimated depending on the availability of either CI, SE, t and *P* values or by calculating a correlation coefficient (Corr) from a known change from baseline standard deviation. A 95 % CI was used to calculate missing sd and considering the absence of studies with sd changes from baseline to follow-up, an extra analysis utilising a Corr value of 0·7 was performed^(27)^.

The statistical heterogeneity between studies was assessed using the overlap of their 95 % CI and expressed as measures of Cochran’s Q (Chi-square test) and I^2^. Data classification as moderately heterogeneous was based on I^2^ from 50 % to 74 %, and highly heterogeneous from 75 % and above^(28)^. Furthermore, sensitivity analysis was performed to evaluate the robustness of the reported statistical results by discounting the effect of confounding factors on outcome measures through a leave-one-out analysis. Studies with a high risk of bias and/or the study with the highest effect size were discounted through a leave-one-out sensitivity analysis. Publication bias was assessed using Begg’s funnel plots and Egger’s linear regression test^(29)^ using R software. Data were meta-analysed and forest plots were drawn using Review Manager (RevMan 5.4.1). A *P* value of < 0·05 was considered statistically significant.

### Subgroup and sensitivity analyses

Subgroup analyses were performed based on Corr equal to 0·7, age, BMI, treatment dose and duration, soy protein and isoflavone co-supplementation, soy protein supplementation during postmenopause, and whey protein supplementation in participants with sarcopenia and pre-frailty. Sensitivity analyses were performed using a leave-one-out analysis, excluding the study with the largest effect size and the study with the highest bias risk.

## Results

### Search results and study characteristics

**Fig. 1.**
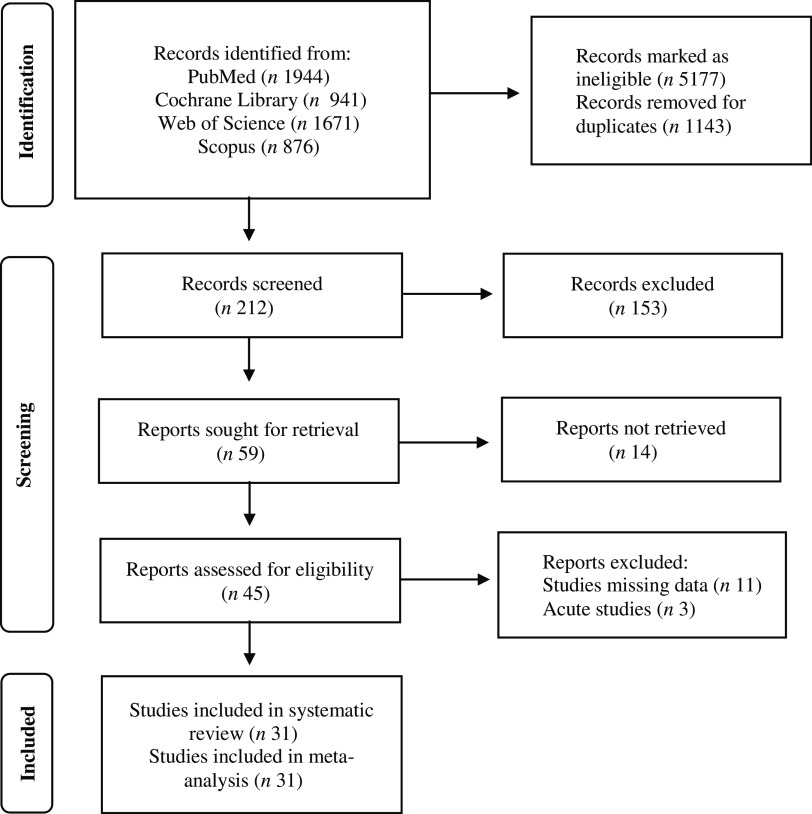
PRISMA flowchart of literature search via databases and registers.

The initial search generated 5432 records, in which 5220 were excluded due to ineligibility issues and study duplicates. Following a full-text review of the remaining 212 studies, 153 articles were removed and 59 articles were sought for retrieval. In total, 45 full-text reports were assessed for eligibility. Acute studies and articles with missing or incomplete data were excluded from the analysis. Overall, 31 studies were included in the systematic review and meta-analysis (Fig. 1).

Study characteristics of the included trials using whey and soy protein supplementation are presented in Table 1 and Table 2, respectively. All trials utilising whey and soy protein supplementation as an intervention in males and females had a mean age between 50 and 80·8 years. Six studies contained additional nutrients alongside whey protein supplementation [one study contained vitamin D^(30)^; one study contained vitamin C and Mg^(31)^, one study contained vitamin D and vitamin E^(32)^, one study contained Zn and Se^(33)^, one study contained medium-chain saturated fatty acids^(34)^, one study contained Ca and vitamin C^(35)^]. In studies providing soy protein supplements, nine out of 13 studies included isoflavones^(35–44)^, one study included phytoestrogens^(45)^ and one study included isoflavones with phytoestrogens^(46)^.

**Table 1.** Characteristics of whey protein supplementation included studies

Study	Country	Study design	Number of participants (M/F)	Age range (years)	Study population	Experimental dose (g/d)	Comparator treatment	Treatment duration	Outcomes
Biesek *et al.* 2021^(35)^	Brazil	Single-blind RCT	E: 11 (0/11) C: 9 (0/9)	E: 73·1 ± 5·3 C: 70·4 ± 3·9	Pre-frailty	21 and 224 mg Ca and 23 mg vitamin C	Placebo	12 weeks	IL-6
Kirk *et al.* 2021^(53)^	UK	Unblinded RCT	E: 23 (11/12) C: 29 (12/17)	E: 71·8 ± 6·5 C: 68·2 ± 5·9	Community-dwelling	1·5 g/kg/body weight	Usual diet (no treatment)	16 weeks	CRP IL-6 TNF-α
Mizubuti *et al.* 2021^(60)^	Brazil	Double-blind RCT	E: 35 (22/13) C: 40 (28/12)	E: 51·6 ± 9·5 C: 52·6 ± 11·4	Chronic liver disease	40	Casein protein	2 weeks	IL-6 TNF-α
Ahmadi *et al.* 2020^(31)^	Iran	Single-blind RCT	E: 23 (23/0) C: 23 (23/0)	E: 62·1 ± 7 C: 63·5 ± 7·2	COPD	16 and vitamin C (685 mg) and Mg (275 mg)	Dietary advice	8 weeks	IL-6 TNF-α
Derosa *et al.* 2020^(47)^	Italy	Single-blind RCT	E: 59 (30/29) C: 58 (29/29)	E: 59·7 ± 9·1 C: 58·6 ± 8·8	Type 2 diabetes	5	Casein protein	3 months	hs-CRP IL-6 TNF-α
Bo *et al.* 2019^(32)^	China	Double-blind RCT	E: 30 (13/17) C: 30 (14/16)	E: 73·2 ± 6·5 C: 74·8 ± 5·9	Sarcopenia	44 and vitamin D (700 IU) and vitamin E (109 mg)	Placebo	6 months	CRP IL-6 TNF-α
Nabuco *et al.* 2019^(55)^	Brazil	Double-blind RCT	E: 13 (0/13) C: 13 (0/13)	E: 68 ± 4·2 C: 70·1 ± 3·9	Sarcopenia	35	Placebo	12 weeks	CRP IL-6 TNF-α
Rakvaag *et al.* 2019^(48)^	Denmark	Double-blind RCT	E: 15 (9/6) C: 16 (8/8)	E: 67 (range: 60–69) C: 62 (range: 58–68)	Abdominal Obesity	72–87	Placebo	12 weeks	hs-CRP TNF-α
Bumrungpert *et al.* 2018^(33)^	Thailand	Double-blind RCT	E: 23 (7/16) C: 19 (3/16)	E: 54·1 ± 9·3 C: 51·5 ± 9·6	Cancer	40 and Zn (2·64 mg) and Se (0·76 mg)	Placebo	12 weeks	hs-CRP
Fernandes *et al.* 2018^(52)^	Brazil	Double-blind RCT	E: 16 (0/16) C: 16 (0/16)	E: 67·3 ± 4·1 C: 67·8 ± 4·0	Healthy	35	Placebo	12 weeks	CRP
Stojkovic *et al.* 2017^(56)^	USA	Double-blind RCT	E: 38 (0/38) C: 46 (0/46)	E: 68·9 ± 0·9 C: 69·3 ± 0·9	Healthy	20	Placebo	18 months	CRP IL-6
Fekete *et al.* 2016^(51)^	Italy	Double-blind Crossover RCT	E: 38 (20/18) C:38 (20/18)	E: 52·9 ± 2·1 C: 52·9 ± 2·1	Prehypertension	56	Placebo	8 weeks	CRP IL-6 TNF-α
Sohrabi *et al.* 2016^(49)^	Iran	Single-blind RCT	E: 23 (10/13) C: 23 (10/13)	E: 57 ± 9·6 C: 55 ± 6·5	Haemodialysis	15	No treatment	8 weeks	hs-CRP IL-6
Rondanelli *et al.* 2016^(30)^	Italy	Double-blind RCT	E: 69 (29/40) C: 61 (24/37)	E: 80·8 ± 6·3 C: 80·2 ± 8·5	Sarcopenia	22 and vitamin D (100 mg)	Placebo	12 weeks	CRP
Bohl *et al.* 2015^(34)^	Denmark	Double-blind RCT	E: 13 (5/8) C: 13 (7/6)	E: 61·1 ± 6 C: 56·7 ± 10·6	Abdominal obesity	60 and MC-SFA (6·9 g)	Casein protein and MC-SFA (6·9 g)	12 weeks	hs-CRP IL-6
Duff *et al.* 2014^(57)^	Canada	Double-blind RCT	E: 21 (8/13) C: 19 (7/12)	E: 57·5 ± 6·3 C: 61·8 ± 4·8	Abdominal obesity	38	Bovine colostrum (62 g/d)	8 weeks	CRP
Weinheimer *et al.* 2012^(59)^	USA	Double-blind RCT	E: 30 C: 84	E: 50 ± 7·1 C: 49 ± 7	Obesity/ Overweight	60	Placebo	18 weeks	CRP
Laviolette *et al.* 2010^(58)^	Canada	Double-blind RCT	E: 12 (10/2) C: 10 (4/6)	E: 62·9 ± 10·1 C: 67·6 ± 4·4	COPD	20	Casein protein	8 weeks	CRP IL-6

E, experimental group; C, comparator group; COPD, chronic obstructive pulmonary disease; CRP, c-reactive protein; F, females; hs-CRP; high sensitivity c-reactive protein; M, males; MC-SFA, medium-chain saturated fatty acids; RCT, randomised controlled trial.

**Table 2.** Characteristics of soy protein supplementation included studies

Study	Country	Study design	N of participants (M/F)	Age range (years)	Health status	Experimental dose (g/d)	Comparator treatment	Treatment duration	Outcomes
Sathyapalan *et al.* 2017^(50)^	Qatar	Double-blind RCT	E: 86 (86/0) C: 85 (85/0)	E: 52 ± 2·5 C: 52 ± 2·5	Type 2 Diabetes	15	Soy protein and isoflavones (66 mg)	3 months	hs-CRP
Liu *et al.* 2012^(41)^	Hong Kong	Double-blind RCT	E: 60 (0/60) C: 60 (0/60)	E: 56·4 ± 4·7 C: 56 ± 4·4	Healthy	15 & isoflavones (100 mg)	Milk protein and isoflavones (100 mg)	6 months	hs-CRP
Ma *et al.* 2011^(54)^	China	Single-blind RCT	E: 45 (14/31) C: 45 (12/33)	E: 51·4 ± 1·7 C: 51·9 ± 1·5	Mild Hypercholesterolemia	18	Milk protein	8 weeks	CRP TNF-α
Sathyapalan *et al.* 2011^(45)^	Qatar	Double-blind crossover RCT	E: 27 (6/21) C: 21 (2/19)	E: 57·2 ± 13·8 C: 57·2 ± 13·8	Subclinical Hypothyroidism	30 and phytoestrogens (2 mg)	Soy protein and phytoestrogens (16 mg)	8 weeks	hs-CRP
Napora *et al.* 2011^(42)^	USA	Double-blind RCT	E: 17 (17/0) C: 16 (16/0)	E: 69·2 ± 2·5 C: 69 ± 2·2	Prostate Cancer	20 and isoflavones (160 mg)	Milk protein	12 weeks	CRP IL-6 TNF-α
Christie *et al.* 2010^(37)^	USA	Double-blind RCT	E: 17 (0/17) C: 16 (0/16)	E: 54·4 ± 3·3 C: 53·3 ± 4·9	Healthy	20 and isoflavones (160 mg)	Placebo	12 weeks	CRP IL-6 TNF-α
Charles *et al.* 2009^(36)^	USA	Double-blind RCT	E: 32 (0/32) C: 43 (0/43)	E: 57·3 ± 1·1 C: 56·1 ± 0·8	Healthy	20 and isoflavones (160 mg)	Whole-milk protein	12 weeks	IL-6 TNF-α
Tormala *et al.* 2009^(44)^	Finland	Single-blind randomised crossover trial	E: 40 (0/40)	E: 57·7 ± 0·8	Healthy	52 & isoflavones (116 mg)	Placebo	8 weeks	CRP
Greany *et al.* 2008^(39)^	USA	Single-blind randomised crossover trial	E: 34 (0/34)	E: 57·7 ± 6	Healthy	26 ± 5 & isoflavones (44 ± 8 mg)	Milk protein	6 weeks	CRP
Fanti *et al.* 2006^(38)^	USA	Double-blind RCT	E: 15 (9/6) C: 10 (6/4)	E: 60·7 ± 3·4 C: 61·6 ± 5·2	End Stage Renal Disease	25 and isoflavones (56 mg)	Milk protein	8 weeks	CRP IL-6 TNF-α
Hanson *et al.* 2006^(46)^	USA	Double-blind RCT	E: 14 (0/14) C: 14 (0/14)	E: 56 (49–70) C: 58 (47–72)	Mild Hypercholesterolemia during	40 and isoflavones (1·2 mg) and phytate (0·22 g)	Soy protein and isoflavones (86 mg) and phytate (0·22 g)	6 weeks	CRP
Hermansen *et al.* 2005^(40)^	Denmark	Double-blind RCT	E: 49 (23/26) C: 51 (19/32)	E: 60·6 ± 3·4 C: 58 ± 4·6	Hypercholesterolemia	30 and isoflavones (100 mg)	Casein protein	24 weeks	TNF-α
Teede *et al.* 2004^(43)^	USA	Double-blind RCT	E: 30 (0/30) C: 20 (0/20)	E: 61 ± 1 C: 62 ± 1	Healthy	40 and isoflavones (118 mg)	Casein protein	3 months	CRP

E, experimental group; C, comparator group; CRP, c-reactive protein; F, females; hs-CRP; high sensitivity c-reactive protein; M, males; RCT, randomised controlled trial.

Furthermore, seven studies measured serum hs-CRP^(33,41,45,47–50)^, 18 studies serum CRP^(30,32,37–39,42–44,46,51–59)^, 16 studies serum IL-6^(31,32,34–38,42,47,49,51,53,55,56,58,60)^ and 14 studies serum TNF-α values^(31,32,36–38,40,42,47,48,51,53–55,60)^. In total, 3274 individuals participated in both groups with 1611 individuals in the intervention group and 1663 individuals in the comparator group (online Supplementary Table 3a–c).

Data collection for whey protein supplementation was performed on three studies in participants with abdominal obesity^(34,48,57)^, three studies with sarcopenia^(30,32,55)^ according to the European Working Group on Sarcopenia in Older People consensus^(61)^, two studies with COPD^(31,58)^, one study with type 2 diabetes^(47)^, pre-frailty^(35)^ based on Fried’s frailty phenotype^(62)^, chronic liver disease^(60)^, cancer^(33)^, haemodialysis^(49)^, prehypertension^(51)^ and obesity^(59)^, while in three studies participants were community-dwelling^(53)^, healthy^(52)^ and on postmenopause^(56)^. Additionally, data collection for soy protein supplementation was performed on seven studies during postmenopause^(36,37,39,41,43,44,46)^, two studies with hypercholesterolaemia^(40,54)^, one study with type 2 diabetes^(50)^, subclinical hypothyroidism^(45)^, prostate cancer^(42)^ and end stage renal disease^(38)^.

### Risk of bias and quality of evidence assessment

Out of 18 studies utilising whey protein supplements, 11 studies had an overall low risk of bias^(20,30,32,35,47–49,51,52,55,57,60)^, five studies had some concerns^(33,34,53,56,58)^ and two studies had a high risk of bias^(31,59)^. Specifically, one study was unblinded^(53)^ and six studies did not provide any details on allocation treatment^(33,34,52,56,58,59)^, whereas although one study claimed there was allocation concealment, no further details were provided^(48)^. In addition, one study had a high risk of trial personnel being aware of participants’ assigned intervention^(31)^. In two studies, there were some concerns regarding missing outcome data^(35,59)^. Finally, in two studies, the outcome assessment could have been influenced by knowledge of the intervention received^(31,53)^.

Out of 13 studies utilising soy protein supplements, nine studies had an overall low risk of bias^(36–38,40–43,45,50)^, one study had some concerns^(46)^ and three studies had a high risk of bias^(39,44,54)^. Particularly, three studies did not provide details on allocation concealment^(39,44,46)^, while two studies claimed there was allocation concealment; however, no further details were provided^(45,50)^. Furthermore, three studies had a high risk of trial personnel being aware of participants’ assigned intervention^(39,44,54)^ and likewise, in three studies, the outcome assessment could have been influenced by knowledge of intervention received^(39,44,54)^.

Traffic light plots were created using robvis visualisation tool. A detailed description of Risk-of-bias 2 traffic light plots for whey and soy protein supplementation studies are presented in Supplementary Tables 4 and 5, respectively. Finally, the GRADE system approach showed that the quality of evidence for the primary outcomes was moderate (Supplementary Tables 6a–d and 7a–d).

### Effect of whey protein supplementation on circulating inflammatory markers analysis

**Fig. 2.**
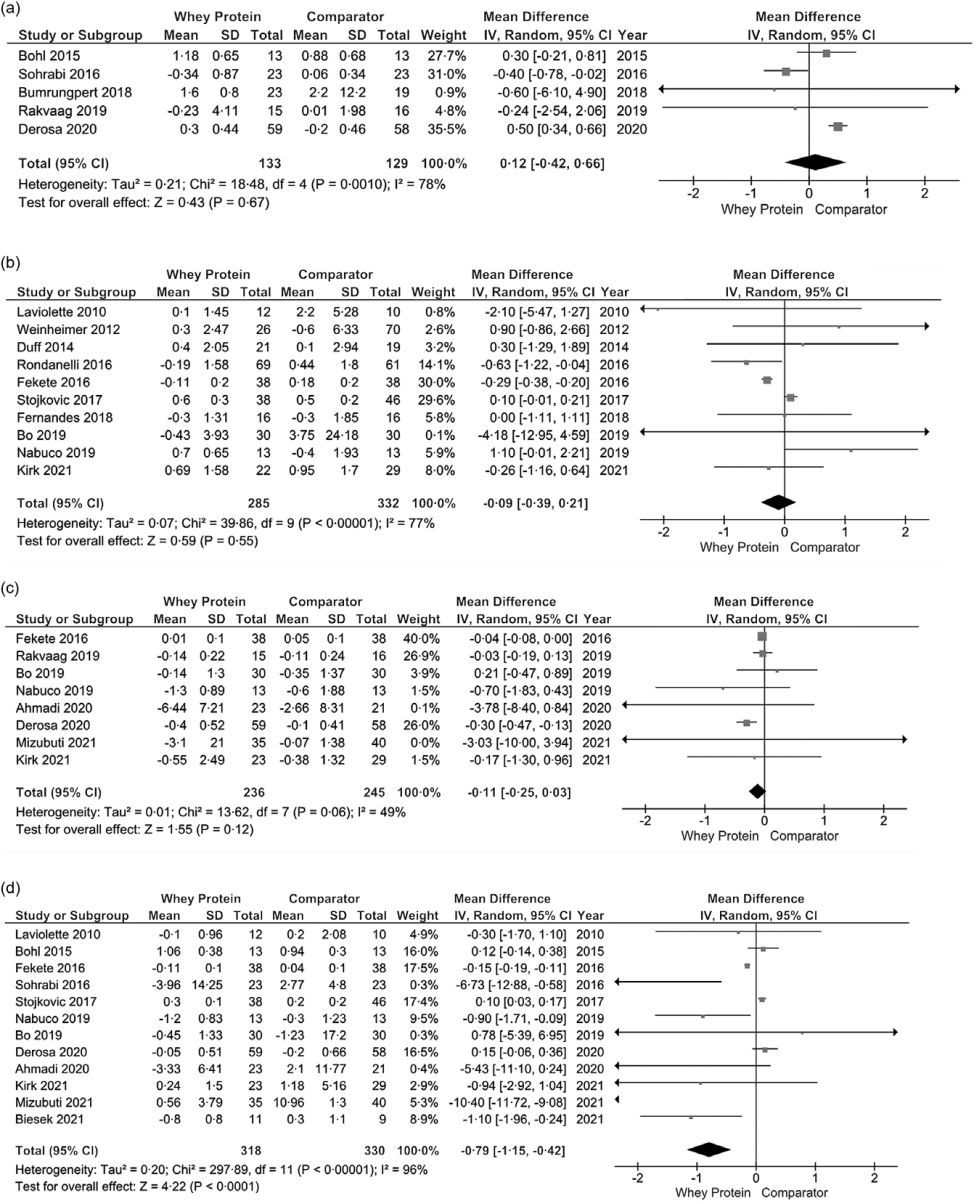
Effects of whey protein supplementation on (a) hs-CRP, (b) CRP, (c) TNF-α and (d) IL-6. CRP, C-reactive protein.

Following whey protein supplementation, no changes were observed on serum hs-CRP (*k* = 5, MD: 0·12, 95 % CI: −0·42, 0·66, I^2^ = 78 %) (Fig. 2a), serum CRP (*k* = 10, MD: −0·09, 95 % CI: −0·39, 0·21, I^2^ = 77 %) (Fig. 2b), and serum TNF-α levels (*k* = 8, MD: −0·11, 95 % CI: −0·25, 0·03, I^2^ = 49 %) (Fig. 2c). Interestingly, whey protein supplementation reduced serum IL-6 levels significantly (*k* = 12, MD: −0·79, 95 % CI: −1·15, −0·42) (Fig. 2d); however, a high heterogeneity among studies was observed (I^2^ = 96 %). Using Corr equal to 0·7 did not demonstrate any significant changes compared with the main analysis (online Supplementary Fig. 1a–d).

### Subgroup analysis of whey protein supplementation trials

Subgroup analysis based on age revealed no significant changes in serum hs-CRP, CRP, TNF-α and IL-6 in adults < 60 and ≥ 60 years of age (online Supplementary Fig. 3a, d).

A subgroup analysis revealed no benefits of whey protein supplementation in individuals with sarcopenia and pre-frailty on serum CRP (*k* = 3, MD: 0·02, 95 % CI: –1·60, 1·65, I^2^ = 75 %) (online Supplementary Fig. 13a) and TNF-α levels (*k* = 2, MD: –0·13 95 % CI: -0·99, 0·73, I^2^ = 45 %) (online Supplementary Fig. 13b); however, whey protein displayed a significant reduction of serum IL-6 levels (*k* = 3, MD: –0·98, 95 % CI: –1·56, –0·39, I^2^ = 0 %) (Supplementary Fig. 13c).

Based on treatment duration, whey protein supplementation ≤ 8 weeks showed a significant reduction in serum CRP levels (*k* = 4, MD: –0·30, 95 % CI: –0·39, –0·21, I^2^ = 0 %) compared with a treatment duration of > 8 weeks (*k* = 6, MD: 0·13, 95 % CI: –0·13, 0·40, I^2^ = 9 %) (online Supplementary Fig. 9a), whereas serum TNF-α and IL-6 concentrations remained unaltered (online Supplementary Fig. 9b, c).

Significant reductions of serum CRP levels were revealed in participants with BMI < 25 kg/m^2^ (*k* = 2, MD: –0·65, 95 % CI: –1·23, 0·06, I^2^ = 0 %) *vs*. BMI ≥ 25 kg/m^2^ (*k* = 8, MD: 0·00, 95 % CI: –0·32, 0·32, I^2^ = 80 %) (online Supplementary Fig. 5a), whereas a significant decline was observed in serum IL-6 levels in participants with BMI ≥ 25 kg/m^2^ (*k* = 7, MD: –1·00, 95 % CI: –1·14, –0·58, I^2^ = 97 %) (online Supplementary Fig. 5c).

In addition, an intervention dose of ≥ 30 g/d led to significant decreases in serum IL-6 levels (*k* = 6, MD: −2·15, 95 % CI: −3·41, 0·89, I^2^ = 96 %) (online Supplementary Fig. 7c), while serum CRP and TNF-α concentrations compared with the comparator group remained statistically unchanged (online Supplementary Fig. 7a, b). All available information regarding subgroup analyses and whey protein supplementation are detailed in Supplementary Table 8a, b.

### Effect of soy protein supplementation on circulating inflammatory markers analysis

**Fig. 3.**
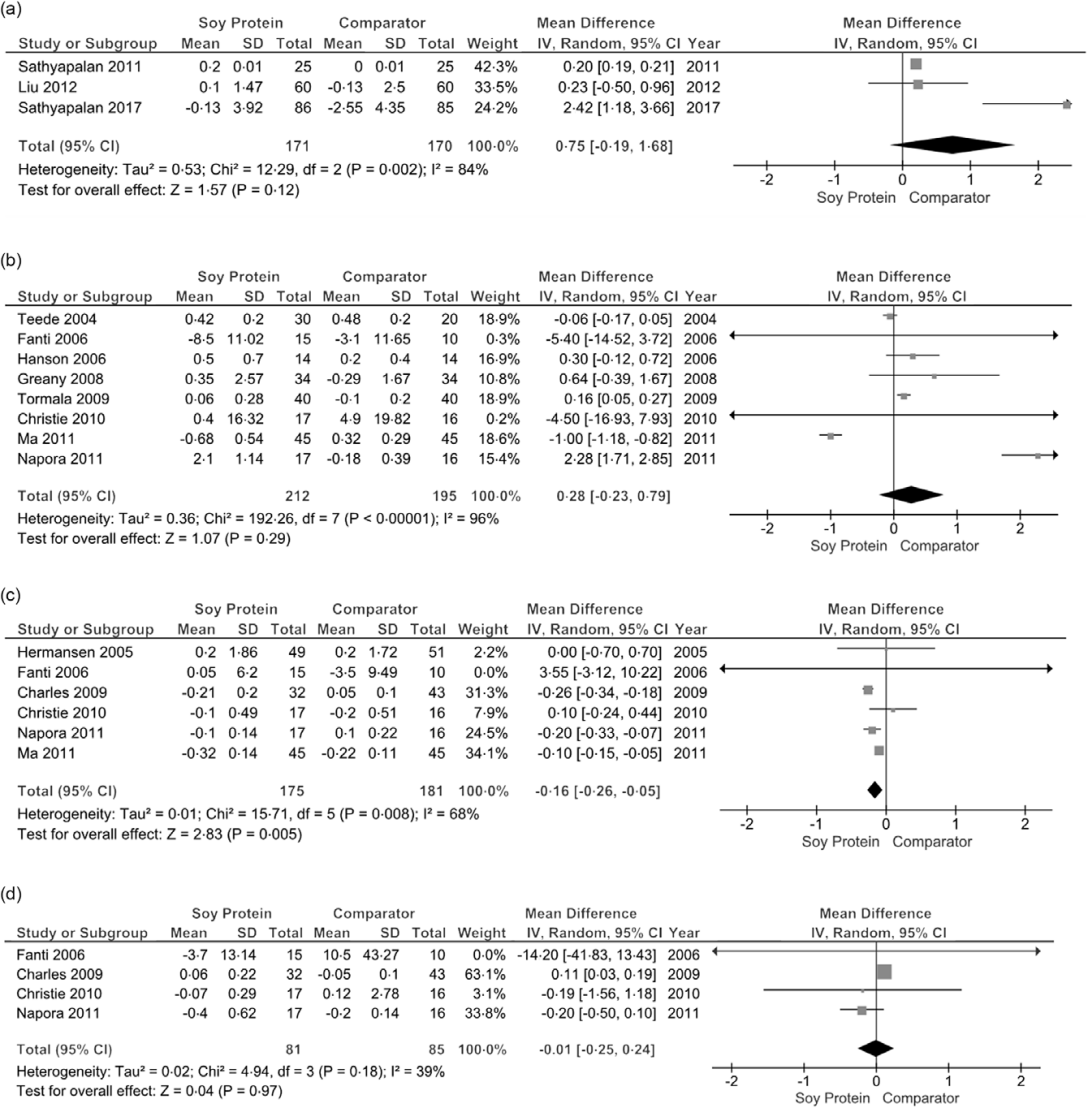
Effects of soy protein supplementation on (a) hs-CRP, (b) CRP, (c) TNF-α and (d) IL-6. CRP, C-reactive protein.

Following soy protein supplementation, no changes were observed on serum hs-CRP (*k* = 3, MD: 0·75, 95 % CI: −0·19, 0·66, *P* = 0·12, I^2^ = 84 %) (Fig. 3a), serum CRP (*k* = 8, MD: 0·28, 95 % CI: −0·23, 0·79, I^2^ = 96 %) (Fig. 3b) and serum IL-6 levels (*k* = 4, MD: −0·01, 95 % CI: −0·25, 0·24, I^2^ = 39 %) (Fig. 3d). Soy protein supplementation however displayed a significant reduction in serum TNF-α (*k* = 6, MD: −0·16, 95 % CI: −0·26, −0·05) (Fig. 3c), which was accompanied by a moderate homogeneity among studies (I^2^ = 68 %).

### Subgroup analysis of soy protein supplementation trials

Using Corr equal to 0·7 did not demonstrate any significant changes compared with the main analysis (online Supplementary Figures 2a–d).

Subgroup analysis based on age revealed a significant reduction of serum TNF-α levels in older adults ≥ 60 years (*k* = 3, MD: –0·19, 95 % CI: –0·32, –0·07, I^2^ = 0 %) as opposed to older adults < 60 years (*k* = 3, MD: –0·14, 95 % CI: –0·29, 0·01, I^2^ = 85 %) (online Supplementary Fig. 4c).

Subgroup analyses showed a significant decline of serum TNF-α levels in older adults with BMI ≥ 25 kg/m^2^ (*k* = 5, MD: –0·20, 95 % CI: –0·31, –0·08, I^2^ = 34 %) (online Supplementary Fig. 6c), soy protein dose < 30 g/d (*k* = 6, MD: –0·16, 95 % CI: –0·26, –0·05, I^2^ = 68 %) (online Supplementary Fig. 8b), treatment duration > 8 weeks (*k* = 4, MD: –0·20, 95 % CI: –0·31, –0·09, I^2^ = 38 %) (online Supplementary Fig. 10c) and addition of isoflavones (*k* = 5, MD: –0·20, 95 % CI: –0·31, –0·08, I^2^ = 34 %) (online Supplementary Fig. 11c). Notably, the addition of isoflavones also demonstrated a significant increase in serum CRP levels (*k* = 7, MD: 0·53, 95 % CI: 0·12, 0·94) (online Supplementary Fig. 11b), although there was a high heterogeneity among trials (I^2^ = 91 %).

There were not enough number of studies for treatment duration and sarcopenia status subgroup analysis with the soy protein supplementation.

### Sensitivity analysis based on effect size and bias risk

Sensitivity analyses using a leave-one-out strategy based on the effect size of whey protein (online Supplementary Fig. 14a–d) and soy protein supplementation studies (online Supplementary Fig. 15a–d) did not alter outcome measures. Likewise, sensitivity analyses using a leave-one-out strategy for bias risk did not reveal any changes compared with the results from the main analysis (whey protein studies: Supplementary Fig. 16a–d; soy protein studies, Supplementary Fig. 17a, b).

### Publication bias

Visual examination to test for asymmetry among studies for serum IL-6 and CRP levels using Begg’s funnel plots are illustrated in Supplementary Fig. 18a, b and Supplement Fig. 18c, d, respectively. Egger’s linear regression test revealed no evidence for publication bias in both the intervention (*z* = −0·6174, *P* = 0·5369) and the comparator group (*z* = −0·0367, *P* = 0·9708) for serum IL-6 levels following whey protein supplementation based on twelve RCT in this meta-analysis. Additionally, Egger’s linear regression test also revealed no evidence for publication bias in the intervention group for serum CRP levels (*z* = −0·0043, *P* = 0·9966); however, an increased risk for publication bias was observed in the comparator group (*z* = 2·5193, *P* = 0·0118).

## Discussion

This meta-analysis showed a significant decline in circulating IL-6 and TNF-α levels following whey and soy protein supplementation, respectively. Subgroup analysis based on age (< 60 years) revealed a significant reduction of serum TNF-α following whey protein consumption, while subgroup analysis accounting for sarcopenia and pre-frailty status also exhibited a significant reduction of serum IL-6. In addition, a decline in serum CRP levels was observed following a treatment duration of ≤ 8 weeks and in participants with BMI ≤ 25 kg/m^2^. Similarly, subgroup analyses based on age (≥ 60 years) and treatment duration of > 8 weeks showed a significant reduction of serum TNF-α following soy protein supplementation, while the addition of isoflavones exhibited further benefits by reducing serum CRP levels. Overall, these findings suggest that whey and soy protein supplementation may exert distinct anti-inflammatory properties, which are dependent on participant physiological characteristics, treatment duration, and addition of isoflavones.

A previous meta-analysis has demonstrated that whey protein may mitigate low-grade inflammation by decreasing serum CRP levels; however, the increased heterogeneity among studies may have influenced such findings^(22)^. Although a high degree of heterogeneity among studies was detected, our analysis revealed a significant effect of whey protein supplementation in reducing serum IL-6 levels. Noteworthy that insignificant results were found in the subgroup analyses on serum TNF-α according to age (< 60 *vs*. ≥ 60 years), BMI (< 25 *vs*. ≥ 25 kg/m^2^) and treatment duration (≤ 8 *vs*. > 8 weeks) on serum CRP levels, our findings should be treated with caution due to the small number of studies. Interestingly, our subgroup analysis revealed significant benefits of whey protein supplementation on sarcopenia and pre-frailty, highlighting a significant decline in circulating IL-6 levels. The combination of these two populations was based on their identical characteristics in relation to muscle mass and strength, displaying a low degree of study heterogeneity. In this context, hospitalised patients with frailty have elicited a beneficial effect on reducing serum IL-6 following whey protein supplementation^(63)^, which may be explained by a concomitant increase in glutathione concentrations and a decrease in ROS accumulation^(64)^. Moreover, reduced serum IL-6 levels have also been demonstrated in individuals with sarcopenia by comparing a whey protein-based product to placebo; however, its nutrient content may have masked the effectiveness of whey protein in isolation^(65)^. Particularly, the combination of carotenoids, choline, vitamin A and E and Fe may exert anti-inflammatory effects^(21,66,67)^ and act as confounders in assessing the efficacy of whey protein in alleviating low-grade inflammation. In a subgroup analysis, one study combined whey protein with vitamin D, which may be partially responsible for serum IL-6 level reduction^(68)^. However, research is conflicting regarding the effects of vitamin D on reducing serum inflammatory markers in older adults^(9,69,70)^. Our findings suggest that a –0·98 pg/ml mean reduction in serum IL-6 concentrations of individuals with sarcopenia and pre-frailty may be of clinical relevance given a 0·7 pg/ml mean difference between younger and older populations based on cross-sectional data^(71)^. Therefore, whey protein supplementation may be a valuable dietary strategy to attenuate the progression of low-grade inflammation and exacerbation of sarcopenia and frailty risk. Considering the increased baseline pro-inflammatory profile in people with sarcopenia, the effects of intact protein supplementation may be more prevalent in these populations. However, due to the limited number of studies and their heterogeneous designs, our results regarding the effectiveness of whey protein in reducing circulating inflammatory markers in individuals with sarcopenia and frailty should be treated with caution.

Previous meta-analyses have revealed that soy-based protein foods and supplements may not alter serum inflammatory status^(72,73)^. However, these findings were based on flavonoid-enriched foods^(65)^ and postmenopausal women from which only serum CRP levels were measured^(72)^. Additionally, experimental studies have not observed a significant effect of soy food consumption on serum CRP levels^(74)^ that may be attributed to the interaction of multiple nutrients contained in whole soy foods^(75)^ compared with isolated sources^(76)^. Our analysis revealed a significant effect of soy protein supplementation in reducing serum TNF-α levels, which are in line with previous research^(77,78)^, although, insignificant reductions of serum IL-6 levels were displayed as reported previously^(23)^. Furthermore, subgroup analysis showed that the addition of isoflavones did not decrease serum CRP and IL-6 levels; however a significant reduction of serum TNF-α was observed. These results may be attributed to the bioactive substances in soy isoflavones (i.e. phenolic compounds, daidzein, and genistein) that exert antioxidant effects^(79,80)^ through glutathione peroxidase regulation and reduction of ROS and malondialdehyde infiltration^(81)^. Although several soy isoflavone doses were administered in this systematic review, subgroup analysis based on dose was not feasible due to the low number of studies. Therefore, whether greater isoflavone quantities correspond to higher decreases of circulating inflammatory cytokines is currently unclear.

### Limitations

Our study was prone to limitations. High variability regarding participant health status, isoflavone dose, and study sample size potentially accounted for the increased heterogeneity in multiple subgroup analyses. The sample size of studies did not allow for subgroup analyses based on healthy populations and individuals with comorbidities. Hence, definitive conclusions around specific conditions and healthy older populations cannot be extrapolated. In addition, the quality of evidence was moderate according to GRADE system approach, while several studies did not use a placebo group as a comparator. Finally, nutrient intake was not controlled in multiple studies, which may have influenced the participants’ inflammatory profile. More importantly, the effects of vitamins, minerals, alcohol, and energy intake may be pivotal contributors in regulating pro-inflammatory cytokine status; hence, the scarcity of data on these parameters should be considered in future studies.

### Conclusions

Systemic low-grade inflammation is a critical contributor to muscle proteolysis during ageing. Our study found a significant reduction of circulating IL-6 and TNF-α levels following whey and soy protein supplementation, respectively. These effects were particularly augmented with the addition of soy isoflavones and populations with sarcopenia and pre-frailty. Whey and soy protein supplementation may serve as a valuable dietary intervention in reducing serum inflammatory cytokine levels, however, more homogeneous studies are required to provide more reliable results on healthy populations and individuals with comorbidities.

## Supplementary material

For supplementary material accompanying this paper visit https://doi.org/10.1017/S0007114522001787. click here to view supplementary material
