# Supplementary material for: Effects of whey and soy protein supplementation on inflammatory cytokines in older adults: a systematic review and meta-analysis
Source: Br J Nutr. 2022 Jun 16;129(5):759–70. doi: 10.1017/S0007114522001787 (PMC9975787; doi:10.1017/S0007114522001787)
Supplement: Supplementary file 1 [file S0007114522001787sup001.zip › S0007114522001787sup0018.docx]

**Supplementary Figure 18.** Begg’s funnel plot of the included studies investigating the effects of whey protein supplementation on (A) serum IL-6 levels in the intervention group, (B) serum IL-6 levels in the comparator group, (C) serum CRP levels in the intervention group, and (D) serum CRP levels in the comparator group.


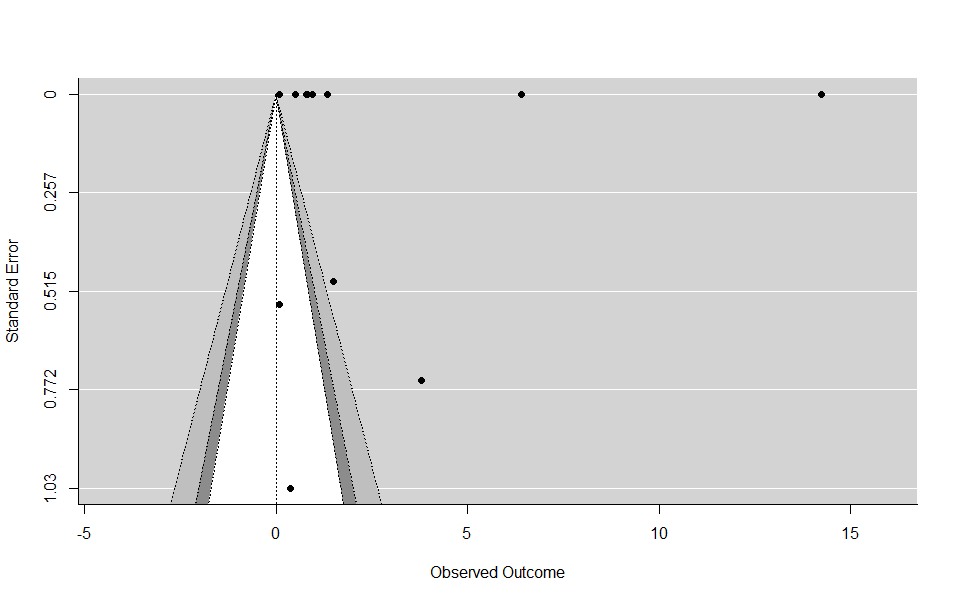


**A**


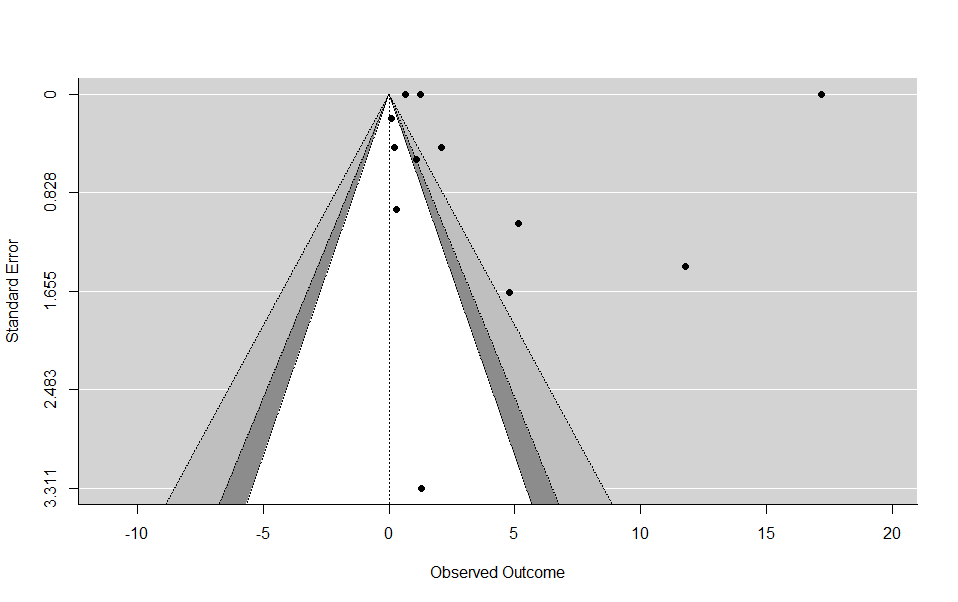


**B**


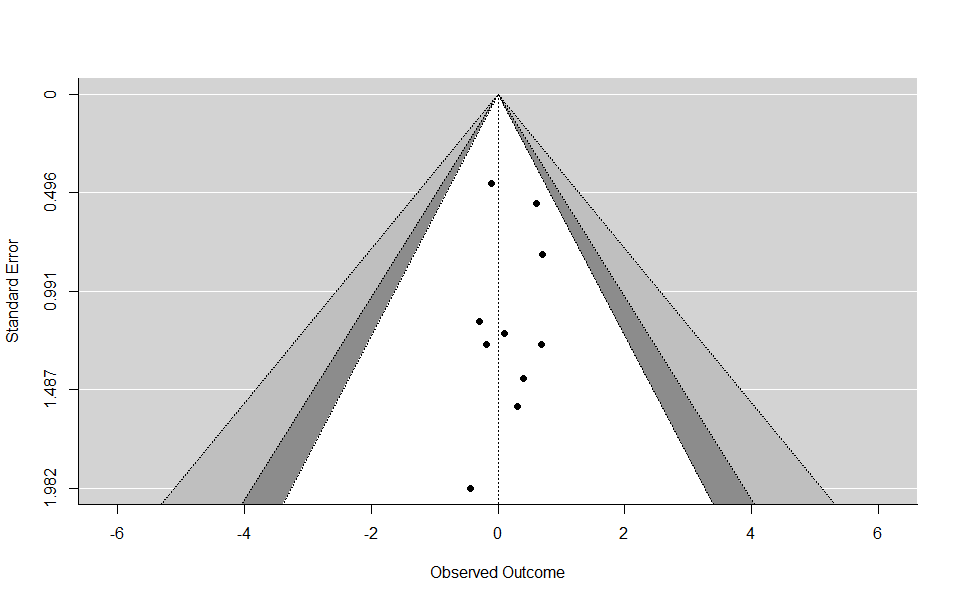


**C**


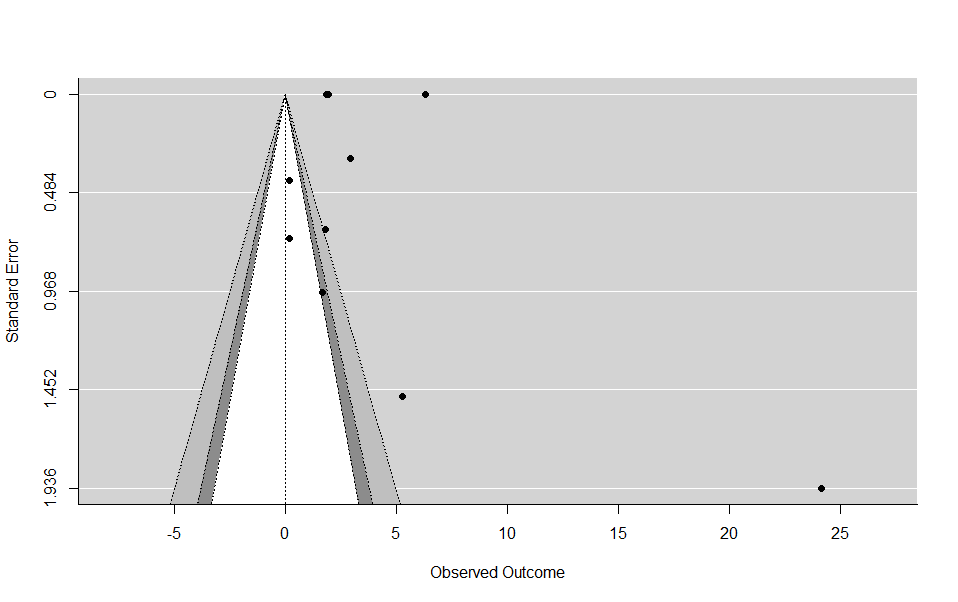


**D**
