# Supplementary material for: Effects of whey and soy protein supplementation on inflammatory cytokines in older adults: a systematic review and meta-analysis
Source: Br J Nutr. 2022 Jun 16;129(5):759–70. doi: 10.1017/S0007114522001787 (PMC9975787; doi:10.1017/S0007114522001787)
Supplement: Supplementary file 1 [file S0007114522001787sup001.zip › S0007114522001787sup0019.docx]

**Supplementary Table 1.** Full search strategy protocol. The mesh terms used for the 4 different electronic databases during the literature search process are provided in the following table.

| **Electronic database** | **Search terms** | **N^o^ of results** |
| --- | --- | --- |
| PubMed | (old OR elderly OR postmenopausal OR ageing populations) AND (soy OR casein OR whey OR protein supplementation) AND (inflammation OR inflammatory markers OR cytokines OR pro-inflammatory OR interleukin OR IL-6 OR C-reactive protein OR high sensitivity c-reactive protein OR CRP OR hs-CRP OR tumor necrosis factor-alpha OR TNF-a) | 1944 |
| Scopus | (old OR elderly OR postmenopausal OR ageing) AND (soy OR casein OR whey OR (protein AND supplementation)) AND (inflammation OR (inflammatory AND markers) OR cytokines OR pro-inflammatory OR interleukin OR IL-6 OR (C-reactive AND protein) OR (high AND sensitivity AND c-reactive protein) OR CRP OR hs-CRP OR (tumor AND necrosis AND factor-alpha) OR TNF-a) | 876 |
| Web of Science | (old OR elderly OR postmenopausal OR ageing populations) AND (soy OR casein OR whey OR protein supplementation) AND (inflammation OR inflammatory markers OR cytokines OR pro-inflammatory OR interleukin OR IL-6 OR C-reactive protein OR high sensitivity c-reactive protein OR CRP OR hs-CRP OR tumor necrosis factor-alpha OR TNF-a) | 1671 |
| Cochrane Library | (old OR elderly OR postmenopausal OR ageing populations) AND (soy OR casein OR whey OR protein supplementation) AND (inflammation OR inflammatory markers OR cytokines OR pro-inflammatory OR interleukin OR IL-6 OR C-reactive protein OR high sensitivity c-reactive protein OR CRP OR hs-CRP OR tumor necrosis factor-alpha OR TNF-a) | 941 |

**Supplementary Table 2.** Inclusion and exclusion criteria for the meta-analysis.

|  | **Inclusion** | **Exclusion** |
| --- | --- | --- |
| **Population** | - Adults aged ≥ 50 years old | - In vivo and in vitro studies - Institutionalized/hospitalized - Pregnancy - Adults aged <50 |
| **Intervention** | - Randomized controlled and crossover trials - Whey protein supplements - Soy protein supplements with or without isoflavones - Appropriate non-identical treatment | - Non-randomized trials - Acute studies (i.e., one-day duration) - Food fortification with whey or soy protein - Whey/soy peptides - Whole foods containing whey/soy protein |
| **Other** | - Articles written in English | - Conference and Review Papers - Book chapters and Surveys - Studies not reporting baseline and follow-up values |

**Supplementary Table 3.** The effect of (A) whey protein supplementation, (B) soy protein supplementation, and (C) soy protein supplementation with isoflavones on inflammatory markers in older adults.

| **Outcome** | **N^o^ of studies** | **Total N^o^ of participants** | **Intervention**  **group size** | **Comparator**  **group size** | **Mean difference**  **(95% CI)** | **I^2^** | **Chi^2^** | **P value (heterogeneity)** | **P value (Effect size)** |
| --- | --- | --- | --- | --- | --- | --- | --- | --- | --- |
| hs-CRP (mg/L) | 6 | 265 | 133 (50.2%) | 129 (49.8%) | 0.12 (-0.42, 0.66) | 78% | 18.48 | 0.001 | 0.67 |
| CRP (mg/L) | 10 | 610 | 285 (46.3%) | 325 (53.7%) | -0.09 (-0.39, 0.21) | 77% | 39.86 | <0.001 | 0.55 |
| IL-6 (pg/mL) | 12 | 648 | 318 (49.1%) | 330 (50.9%) | -0.79 (-1.15, -0.42) | 96% | 297.89 | <0.001 | <0.01 |
| TNF-a (pg/mL) | 8 | 481 | 236 (49.1%) | 245 (50.9%) | -0.11 (-0.25, 0.03) | 49% | 13.62 | 0.06 | 0.12 |

*P<0.05 indicates statistical significance

**A**

**B**

| **Outcome** | **N^o^ of studies** | **Total N^o^ of participants** | **Intervention**  **group size** | **Comparator**  **group size** | **Mean difference**  **(95% CI)** | **I^2^** | **Chi^2^** | **P value (heterogeneity)** | **P value (Effect size)** |
| --- | --- | --- | --- | --- | --- | --- | --- | --- | --- |
| hs-CRP (mg/L) | 3 | 341 | 171 (50.1%) | 170 (49.9%) | 0.75 (-0.19, 1.68) | 84% | 12.29 | 0.002 | 0.12 |
| CRP (mg/L) | 8 | 407 | 212 (52.1%) | 195 (47.9%) | 0.28 (-0.23, 0.79) | 96% | 192.26 | <0.001 | 0.29 |
| IL-6 (pg/mL) | 4 | 166 | 81 (48.9%) | 85 (51.1%) | -0.01 (-0.25, 0.24) | 39% | 4.94 | 0.18 | 0.97 |
| TNF-a (pg/mL) | 6 | 356 | 175 (49.2%) | 181 (50.8%) | -0.16 (-0.26, -0.05) | 68% | 15.71 | 0.008 | <0.01 |

*P<0.05 indicates statistical significance

**C**

| **Outcome** | **N^o^ of studies** | **Total N^o^ of participants** | **Intervention**  **group size** | **Comparator**  **group size** | **Mean difference**  **(95% CI)** | **I^2^** | **Chi^2^** | **P value (heterogeneity)** | **P value (Effect size)** |
| --- | --- | --- | --- | --- | --- | --- | --- | --- | --- |
| hs-CRP (mg/L) | 2 | 219 | 113 (51.6%) | 106 (48.4%) | 1.49 (-0.08, 3.06) | 85% | 6.54 | 0.01 | 0.06 |
| CRP (μg/L) | 7 | 317 | 167 (52.7%) | 150 (47.3%) | 0.53 (0.12, 0.94) | 91% | 68.01 | <0.001 | 0.01* |
| IL-6 (pg/mL) | 4 | 166 | 81 (48.9%) | 86 (51.1%) | -0.01 (-0.25, 0.24) | 39% | 4.94 | 0.18 | 0.97 |
| TNF-a (pg/mL) | 5 | 266 | 130 (48.9%) | 136 (51.1%) | -0.20 (0.31, -0.08) | 34% | 6.05 | 0.20 | 0.0006* |

*P<0.05 indicates statistical significance

**Supplementary Table 4.** Quality of evidence through Risk of Bias 2 (Rob2) tool for studies using whey protein


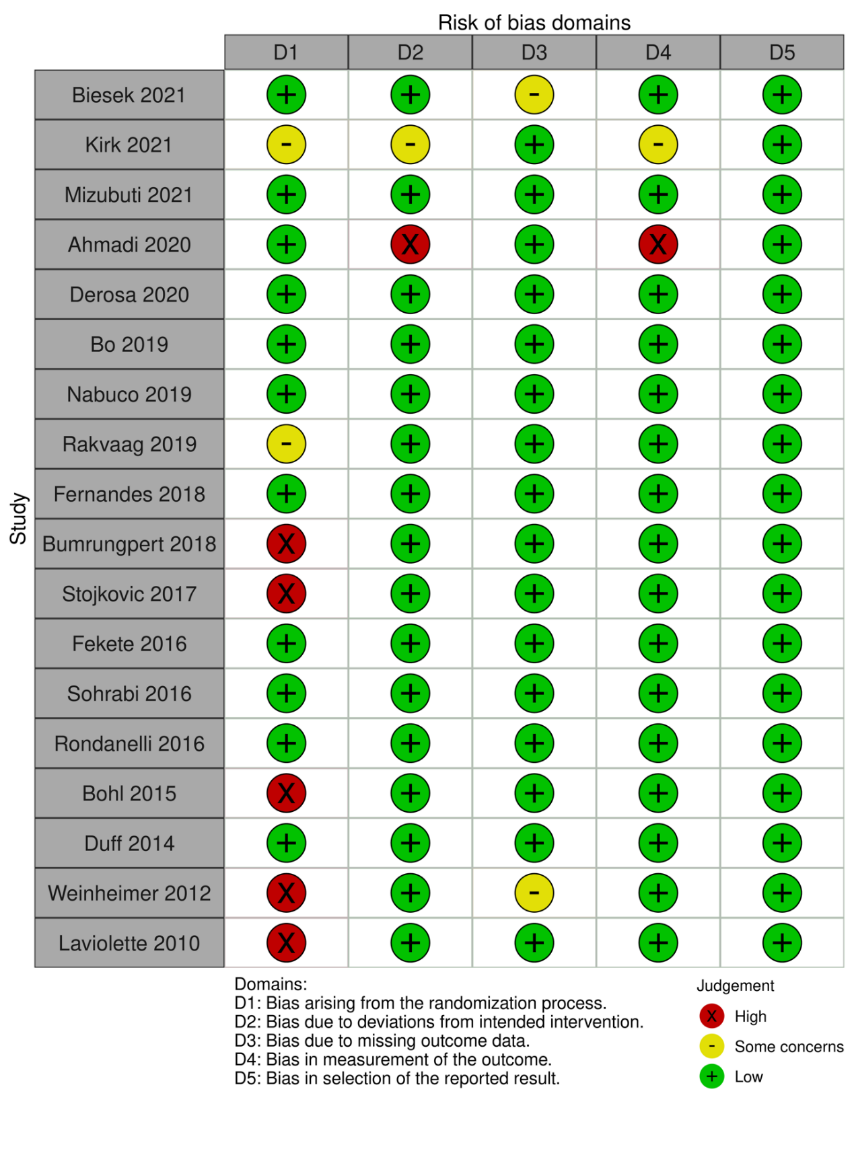


**Supplementary Table 5.** Quality of evidence through Risk of Bias 2 (Rob2) tool for studies using soy protein.


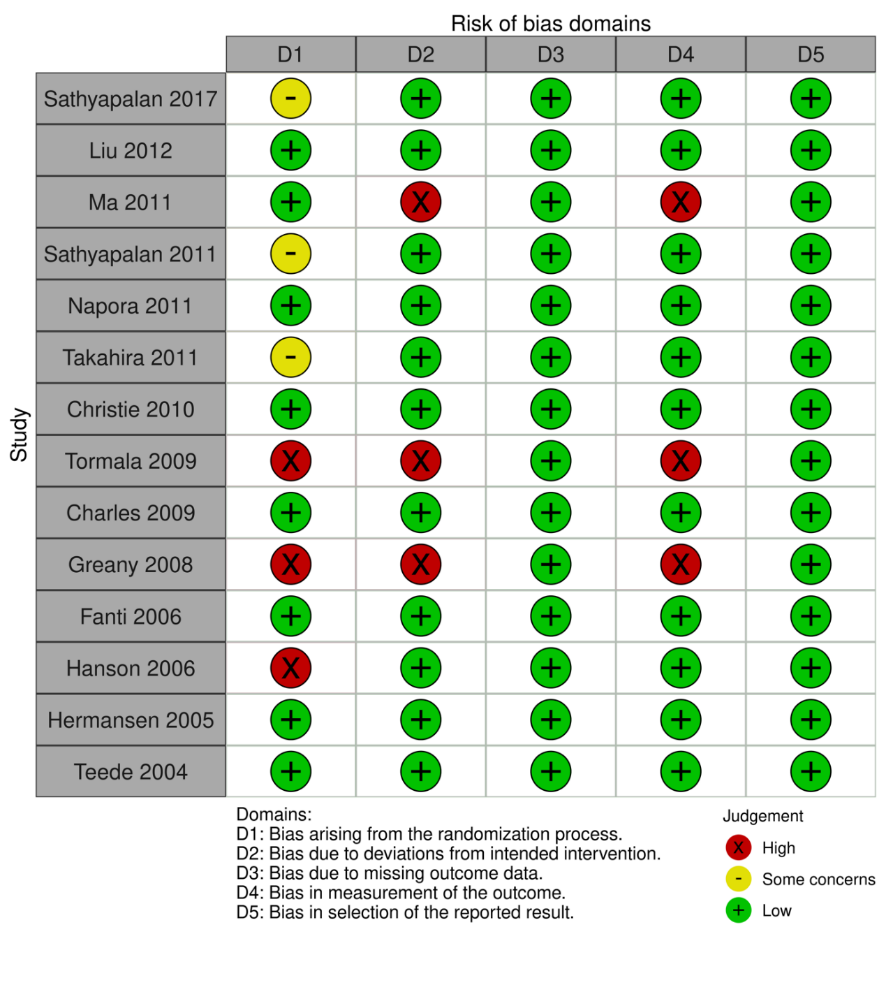


**Supplementary Table 6.** GRADE criteria on whey protein supplementation and serum (A) hs-CRP, (B) CRP, (C) TNF-a, and (D) IL-6 levels.

**A**

| **Certainty assessment** | | | | | | | **№ of patients** | | **Effect** | | **Certainty** | **Importance** |
| --- | --- | --- | --- | --- | --- | --- | --- | --- | --- | --- | --- | --- |
| **№ of studies** | **Study design** | **Risk of bias** | **Inconsistency** | **Indirectness** | **Imprecision** | **Other considerations** | **Whey Protein** | **Comparator** | **Relative (95% CI)** | **Absolute (95% CI)** |  |  |
|  | | | | | | | | | | | | |
| 5 | Randomized trials | Not serious | Not serious | Not serious | Not serious | - | 133 | 129 | - | MD 0.12  (-0.42, 0.66) | ⨁⨁⨁⨁ High | 3-Not Important |

**CI:** confidence interval; **MD:** mean difference

| **Certainty assessment** | | | | | | | **№ of patients** | | **Effect** | | **Certainty** | **Importance** |
| --- | --- | --- | --- | --- | --- | --- | --- | --- | --- | --- | --- | --- |
| **№ of studies** | **Study design** | **Risk of bias** | **Inconsistency** | **Indirectness** | **Imprecision** | **Other considerations** | **Whey protein** | **Comparator** | **Relative (95% CI)** | **Absolute (95% CI)** |  |  |
|  | | | | | | | | | | | | |
| 10 | Randomized trials | Not serious | Not serious | Not serious | Serious^a^ | - | 285 | 332 | - | MD -0.09 (-0.39,0.21) | ⨁⨁⨁◯ Moderate | 3-Not Important |

**CI:** confidence interval; **MD:** mean difference; **Explanations**, **a.** The RCT from Bo et al 2019 has very high ΔSD values that are not relatively in line with the rest of the studies. However, it only accounts for an effect size of 0.4% in our analysis.

**B**

| **Certainty assessment** | | | | | | | **№ of patients** | | **Effect** | | **Certainty** | **Importance** |
| --- | --- | --- | --- | --- | --- | --- | --- | --- | --- | --- | --- | --- |
| **№ of studies** | **Study design** | **Risk of bias** | **Inconsistency** | **Indirectness** | **Imprecision** | **Other considerations** | **Whey protein** | **Comparator** | **Relative (95% CI)** | **Absolute (95% CI)** |  |  |
|  | | | | | | | | | | | | |
| 8 | Randomized trials | Serious^a^ | Not serious | Not serious | Not serious | - | 236 | 245 | - | MD -0.11 (-0.25, 0.03) | ⨁⨁⨁◯ Moderate | 4-Not Important |

**CI:** confidence interval; **MD:** mean difference; **Explanations**, **a.** The trial personnel in the Ahmadi et al 2020 RCT could be aware of participants' assigned intervention and the outcome assessment could be influenced by knowledge of intervention received.

**C**

| **Certainty assessment** | | | | | | | **№ of patients** | | **Effect** | | **Certainty** | **Importance** |
| --- | --- | --- | --- | --- | --- | --- | --- | --- | --- | --- | --- | --- |
| **№ of studies** | **Study design** | **Risk of bias** | **Inconsistency** | **Indirectness** | **Imprecision** | **Other considerations** | **Whey protein** | **Comparator** | **Relative (95% CI)** | **Absolute (95% CI)** |  |  |
|  | | | | | | | | | | | | |
| 12 | Randomized trials | Not serious | Not serious | Not serious | Not serious | - | 318 | 330 | - | MD -0.79 (-1.15, -0.42) | ⨁⨁⨁⨁ High | 6-Important |

**CI:** confidence interval; **MD:** mean difference

**D**

**Supplementary Table 7.** GRADE criteria on soy protein supplementation and serum (A) hs-CRP, (B) CRP, (C) TNF-a, and (D) IL-6 levels.

**A**

| **Certainty assessment** | | | | | | | **№ of patients** | | **Effect** | | **Certainty** | **Importance** |
| --- | --- | --- | --- | --- | --- | --- | --- | --- | --- | --- | --- | --- |
| **№ of studies** | **Study design** | **Risk of bias** | **Inconsistency** | **Indirectness** | **Imprecision** | **Other considerations** | **Soy Protein** | **Comparator** | **Relative (95% CI)** | **Absolute (95% CI)** |  |  |
|  | | | | | | | | | | | | |
| 3 | Randomized trials | Not serious | Not serious | Not serious | Not serious | - | 171 | 170 | - | MD 0.75 (0.19, 1.68) | ⨁⨁⨁⨁ High | 4-Important |

**CI:** confidence interval; **MD:** mean difference

| **Certainty assessment** | | | | | | | **№ of patients** | | **Effect** | | **Certainty** | **Importance** |
| --- | --- | --- | --- | --- | --- | --- | --- | --- | --- | --- | --- | --- |
| **№ of studies** | **Study design** | **Risk of bias** | **Inconsistency** | **Indirectness** | **Imprecision** | **Other considerations** | **Soy Protein** | **Comparator** | **Relative (95% CI)** | **Absolute (95% CI)** |  |  |
|  | | | | | | | | | | | | |
| 8 | Randomized trials | Serious^a^ | Serious^b^ | Not serious | Not serious | - | 212 | 195 | - | MD 0.28  (-0.23, 0.79) | ⨁⨁◯◯ Low | 4-Important |

**CI:** confidence interval; **MD:** mean difference; **Explanations**, **a.** Three RCTs (Ma, Tormala, Greany) had a high risk of bias, considering that trial personnel could be aware of the assigned intervention and there was no information related to allocation concealment; **b.** The RCTs, Fanti et al 2006 and Christie et al 2010 has inconsistent ΔSD values in both the comparator and intervention group, however, they accounted for an effect size of 0.5% combined in the analysis.

**B**

| **Certainty assessment** | | | | | | | **№ of patients** | | **Effect** | | **Certainty** | **Importance** |
| --- | --- | --- | --- | --- | --- | --- | --- | --- | --- | --- | --- | --- |
| **№ of studies** | **Study design** | **Risk of bias** | **Inconsistency** | **Indirectness** | **Imprecision** | **Other considerations** | **Soy Protein** | **Comparator** | **Relative (95% CI)** | **Absolute (95% CI)** |  |  |
|  | | | | | | | | | | | | |
| 6 | Randomized trials | Not serious | Serious^a^ | Not serious | Not serious | - | 175 | 181 | - | MD -0.16 (-0.26, 0.05) | ⨁⨁⨁◯ Moderate | 4-Important |

**CI:** confidence interval; **MD:** mean difference; **Explanations**, **a.** The RCTs, Fanti et al 2006 and Christie et al 2010 has inconsistent ΔSD values in both the comparator and intervention group, however, they accounted for an effect size of 7.9% combined in the analysis.

**C**

| **Certainty assessment** | | | | | | | **№ of patients** | | **Effect** | | **Certainty** | **Importance** |
| --- | --- | --- | --- | --- | --- | --- | --- | --- | --- | --- | --- | --- |
| **№ of studies** | **Study design** | **Risk of bias** | **Inconsistency** | **Indirectness** | **Imprecision** | **Other considerations** | **Soy Protein** | **Comparator** | **Relative (95% CI)** | **Absolute (95% CI)** |  |  |
|  | | | | | | | | | | | | |
| 4 | Randomized trials | Not serious | Not serious | Not serious | Not serious | - | 81 | 85 | - | MD -0.01  (-0.25, 0.24) | ⨁⨁⨁⨁ High | 2-Not Important |

**CI:** confidence interval; **MD:** mean difference

**D**

**Supplementary Table 8.** Subgroup analyses for studies evaluating the effect of (A) whey protein and (B) soy protein supplementation on inflammatory markers in older adults.

**A**

|  | Subgroup | No of  trials | Change in  hs-CRP  (95% CI) | Change in  CRP (95% CI) | Change in  TNF-a (95% CI) | Change in  IL-6 (95% CI) | P-value | I^2^ |
| --- | --- | --- | --- | --- | --- | --- | --- | --- |
| Age | < 60 years  ≥ 60 years | hs-CRP (k = 3) CRP (k = 3)  TNF-a (k = 2) IL-6 (k = 3)  hs-CRP (k = 2) CRP (k = 7)  TNF-a (k = 6) IL-6 (k = 9) | 0.05  (-0.79, 0.90)  0.27  (-0.22, 0.77) | -0.19 (-0.61, 0.23)  -0.07 (-0.52, 0.39) | -3.55  (-7.40, -0.30)  -0.11 (-0.24, 0.03) | -5.59  (-14.12, 2.94)  -0.12 (-0.33, 0.09) | hs-CRP: 0.90 CRP: 0.38 TNF-a: 0.07 IL-6: 0.20  hs-CRP: 0.28 CRP: 0.77 TNF-a: 0.12 IL-6: 0.26 | hs-CRP: 89% CRP: 12% TNF-a: 0% IL-6: 99%  hs-CRP: 0% CRP: 50% TNF-a: 52% IL-6: 85% |
| BMI | < 25 kg/m2  ≥ 25 kg/m2 | hs-CRP (k = 3) CRP (k = 2)  TNF-a (k = 3) IL-6 (k = 3)  hs-CRP (k = 2) CRP (k = 8)  TNF-a (k = 5) IL-6 (k = 7) | 0.05 (-0.79, 0.90)  0.27 (-0.22, 0.77) | -0.65 (-1.23, -0.06)  0.00 (-0.32, 0.32) | -0.17 (-0.77, 0.42)  -0.04 (-0.08, 0.00) | -0.86 (-3.94, 2.23)  -1.00 (-1.41, -0.58) | hs-CRP: 0.90  CRP: 0.03 TNF-a: 0.57 IL-6: 0.59  hs-CRP: 0.28 CRP: 0.98 TNF-a: 0.07 IL-6: <0.01 | hs-CRP: 89% CRP: 0% TNF-a: 53% IL-6: 47%  hs-CRP: 0% CRP: 80% TNF-a: 0% IL-6: 97% |
| Intervention duration | ≤ 8 weeks  > 8 weeks | CRP (k = 4)  TNF-a (k = 3) IL-6 (k = 4)  CRP (k = 6)  TNF-a (k = 5) IL-6 (k = 8) | -  - | -0.30 (-0.39, -0.21)  0.13 (-0.13, 0.40) | -1.13 (-3.63, 1.37)  -0.15 (-0.36, 0.06) | -5.68 (-12.43, 1.07)  -0.05 (-0.26, 0.16) | CRP: <0.01  TNF-a: 0.37 IL-6: 0.10  CRP: 0.32 TNF-a: 0.16 IL-6: 0.65 | CRP: 0% TNF-a: 38% IL-6: 97%  CRP: 9% TNF-a: 44% IL-6: 58% |
| Protein dose | < 30 g/d  ≥ 30 g/d | CRP (k = 3) TNF-a (k = 2) IL-6 (k = 6)  CRP (k = 7)  TNF-a (k = 6) IL-6 (k = 6) | -  - | -0.29 (-1.01, 0.43)  0.03 (-0.43, 0.49) | -1.24 (-4.28, 1.79)  -0.04 (-0.08, 0.00) | -0.08 (-0.43, 0.26)  -2.15 (-3.41, -0.89) | CRP: 0.43 TNF-a: 0.42 IL-6: 0.63  CRP: 0.91 TNF-a: 0.07 IL-6: <0.01 | CRP: 73% TNF-a: 54% IL-6: 69%  CRP: 35%  TNF-a: 0%  IL-6: 98% |
| Health status | Sarcopenia and  Pre-frailty | CRP (k = 3)  TNF-a (k = 2)  IL-6 (k = 3) | - | 0.02 (-1.60, 1.65) | -0.13 (-0.99, 0.73) | -0.98 (-1.56, -0.39) | CRP: 0.98 TNF-a: 0.77 IL-6: 0.001 | CRP: 75% TNF-a: 45% IL-6: 0% |
| Corr | 0.7 | hs-CRP (k = 5) CRP (k = 10)  TNF-a (k = 8) IL-6 (k = 12) | 0.12 (-0.42, 0.66) | -0.06 (-0.36, 0.25) | -0.11 (-0.28, 0.05) | -0.65  (-0.99, -0.30) | hs-CRP: 0.67 CRP: 0.72 TNF-a: 0.17 IL-6: 0.0002 | hs-CRP: 78% CRP: 79% TNF-a: 46% IL-6: 96% |

BMI, body mass index; Corr, correlation coefficient; CRP, c-reactive protein; hs-CRP, high-sensitivity c-reactive protein; IL-6, interleukin-6; TNF-a, tumor necrosis-alpha

**B**

|  | Subgroup | N^o^ of  trials | Change in  hs-CRP  (95% CI) | Change in  CRP (95% CI) | Change in  TNF-a (95% CI) | Change in  IL-6 (95% CI) | P-value | I^2^ |
| --- | --- | --- | --- | --- | --- | --- | --- | --- |
| Age | < 60 years  ≥ 60 years | hs-CRP (*k* = 3) CRP (*k* = 5)  TNF-a (*k* = 3)  IL-6 (*k* = 2)  CRP (*k* = 3) TNF-a (*k* = 3) IL-6 (*k* = 2) | 0.75 (-0.19, 1.68)  - | -0.05 (-0.83, 0.73)  0.75 (-1.49, 2.98) | -0.14 (-0.29, 0.01)  -0.19 (-0.32, -0.07) | 0.11 (0.03, 0.19)  -0.20 (-0.50, 0.10) | hs-CRP: 0.12 CRP: 0.90 TNF-a: 0.06  IL-6: 0.009  CRP: 0.51 TNF-a: 0.002 IL-6: 0.19 | hs-CRP: 84% CRP: 97% TNF-a: 85%  IL-6: 0%  CRP: 97% TNF-a: 0% IL-6: 0% |
| BMI | < 25 kg/m^2^  ≥ 25 kg/m^2^ | CRP (*k* = 2)    hs-CRP (*k* = 2) CRP (*k* = 6)  TNF-a (*k* = 5) IL-6 (*k* = 4) | -  1.22 (-0.95, 3.39) | -0.42 (-1.55, 0.72)  0.67 (-0.29, 1.64) | -  -0.20 (-0.31, -0.08) | -  -0.01 (-0.25, 0.24) | CRP: 0.47  hs-CRP: 0.27 CRP: 0.17 TNF-a: 0.0006 IL-6: 0.97 | CRP: 99%  hs-CRP: 92% CRP: 92% TNF-a: 34% IL-6: 39% |
| Intervention duration | ≤ 8 weeks  > 8 weeks | CRP (*k* = 4)  TNF-a (*k* = 2)  CRP (*k* = 4)  TNF-a (*k* = 4) IL-6 (*k* = 3) | -  - | -0.03 (-0.81, 0.75)  0.92 (-1.33, 3.17) | 0.14 (-1.63, 1.91)  -0.20 (-0.31, -0.09) | -  -0.00 (-0.25, 0.24) | CRP: 0.93  TNF-a: 0.88  CRP: 0.42 TNF-a: 0.0003 IL-6: 0.98 | CRP: 98% TNF-a: 13%  CRP: 97% TNF-a: 38% IL-6: 49% |
| Protein dose | < 30 g/d  ≥ 30 g/d | CRP (k = 5) TNF-a (k = 6) IL-6 (*k* = 4)  CRP (*k* = 3) | -  - | 0.17 (-2.10, 2.43)  0.09 (-0.11, 0.28) | -0.16 (-0.26, -0.05)  - | -0.01 (-0.25, 0.24)  - | CRP: 0.89 TNF-a: 0.005 IL-6: 0.97  CRP: 0.38 | CRP: 97% TNF-a: 68% IL-6: 39%  CRP: 78% |
| Status | Postmenopause | CRP (*k* = 5)  TNF-a (*k* = 2) | - | 0.10 (-0.08, 0.29) | -0.12 (-0.46, 0.22) | - | CRP: 0.28 TNF-a: 0.49 | CRP: 62% TNF-a: 75% |
| Additional nutrients | Soy protein & Isoflavones | hs-CRP (*k* = 2) CRP (*k* = 7) TNF-a (*k* = 5) IL-6 (*k* = 4) | 1.49 (-0.08, 3.06) | 0.53 (0.12, 0.94) | -0.20 (-0.31, -0.08) | -0.01 (-0.25, 0.24) | hs-CRP: 0.06  CRP: 0.01 TNF-a: 0.0006  IL-6: 0.97 | hs-CRP: 85%  CRP: 91% TNF-a: 34%  IL-6: 39% |
| Corr | 0.7 | hs-CRP (*k* = 3) CRP (*k* = 8) TNF-a (*k* = 5) IL-6 (*k* = 4) | 0.75 (-0.19, 1.68) | 0.26 (-0.26, 0.78) | -0.16 (-0.26, -0.05) | -0.01 (-0.25, 0.24) | hs-CRP: 0.12 CRP: 0.32 TNF-a: 0.005 IL-6: 0.97 | hs-CRP: 84% CRP: 96% TNF-a: 68% IL-6: 39% |

BMI, body mass index; Corr, correlation coefficient; CRP, c-reactive protein; hs-CRP, high-sensitivity c-reactive protein; IL-6, interleukin-6; TNF-a, tumor necrosis-alpha
