# Supplementary material for: Effects of whey and soy protein supplementation on inflammatory cytokines in older adults: a systematic review and meta-analysis
Source: Br J Nutr. 2022 Jun 16;129(5):759–70. doi: 10.1017/S0007114522001787 (PMC9975787; doi:10.1017/S0007114522001787)
Supplement: Supplementary file 1 [file S0007114522001787sup001.zip › S0007114522001787sup004.docx]

**Supplementary Figure 4.** Effects of soy protein supplementation on (A) hs-CRP, (B) CRP, (C) TNF-a, and (D) IL-6 based on age (<60 years, ≥60 years).

**B**

**A**

**C**

**D**
