# Supplementary material for: Effects of whey and soy protein supplementation on inflammatory cytokines in older adults: a systematic review and meta-analysis
Source: Br J Nutr. 2022 Jun 16;129(5):759–70. doi: 10.1017/S0007114522001787 (PMC9975787; doi:10.1017/S0007114522001787)
Supplement: Supplementary file 1 [file S0007114522001787sup001.zip › S0007114522001787sup008.docx]

**Supplementary Figure 8.** Effects of soy protein supplementation dose (<30 g/d, ≥30 g/d) on (A) CRP, (B) TNF-a, and (C) IL-6.

**A**

**C**

**B**
